# Residual Cognitive Capacities in Patients With Cognitive Motor Dissociation, and Their Implications for Well-Being

**DOI:** 10.1093/jmp/jhab026

**Published:** 2021-10-16

**Authors:** Mackenzie Graham

**Affiliations:** Oxford University, Oxford, UK

**Keywords:** CMD, cognitive motor dissociation, neuroimaging, well-being

## Abstract

Patients with severe disorders of consciousness are thought to be unaware of themselves or their environment. However, research suggests that a minority of patients diagnosed as having a disorder of consciousness remain aware. These patients, designated as having “cognitive motor dissociation” (CMD), can demonstrate awareness by imagining specific tasks, which generates brain activity detectable via functional neuroimaging. The discovery of consciousness in these patients raises difficult questions about their well-being, and it has been argued that it would be better for these patients if they were allowed to die. Conversely, I argue that CMD patients may have a much higher level of well-being than is generally acknowledged. It is far from clear that their lives are not worth living, because there are still significant gaps in our understanding of how these patients experience the world. I attempt to fill these gaps, by analyzing the neuroscientific research that has taken place with these patients to date. Having generated as comprehensive a picture as possible of the capacities of CMD patients, I examine this picture through the lens of traditional philosophical theories of well-being. I conclude that the presumption that CMD patients do not have lives worth living is not adequately supported.

## I. INTRODUCTION

In 2006, Owen et al. (2006) demonstrated for the first time that a patient who had been repeatedly diagnosed as being in a vegetative state (VS) was actually aware. Since that time, numerous studies have found that a minority of patients diagnosed as being in a VS—a diagnostic term also called “unresponsive wakefulness syndrome” (UWS)— remain covertly aware (Monti et al., 2010; Bardin et al., 2011; Cruse et al., 2011; Chennu et al., 2013; Fernandez-Espejo and Owen, 2013; Naci and Owen, 2013; Stender et al., 2014). In 2015, Schiff coined the term “cognitive motor dissociation” (CMD) to refer to this patient population: behaviorally non-responsive at the bedside, but nevertheless capable of demonstrating awareness using functional neuroimaging (Schiff, 2015).

In addition to its scientific importance, the recognition of awareness in this patient population has important ethical implications. Given that they are conscious, how are these patients faring? Are they suffering? Is it in their best interests to continue living?

There is disagreement in the philosophy of well-being literature about what has “prudential value,” that is, what kinds of things make my life go well, for me (Fletcher, 2016). Experiential accounts (e.g., hedonism) ground well-being in the subjective quality of my experiences, while desire theories hold that well-being is determined by the extent to which my desires are satisfied. Other theories of well-being understand prudential value as independent of my desires. Objective-list theories hold that there are certain goods whose prudential value does not depend on my attitudes toward them, while perfectionist theories argue that what is good for me are those things that help to develop and perfect my nature.

Despite this lack of consensus, however, the prevailing intuition among philosophers (Kahane and Savulescu, 2009; Kitzinger and Kitzinger, 2013; Hawkins, 2016), healthcare practitioners (Demertzi et al., 2011), and members of the general public (Gipson et al., 2014) is that continued existence in a state like CMD is a paradigm case of a life that is not worth living.^1^ The assumption seems to be that these patients are suffering tremendously in their current condition, and any benefits they might accrue from continued existence are not enough to offset this potential suffering. Accordingly, it has been argued that it is in the best interests of these patients to be allowed to die (Kahane and Savulescu, 2009; Hawkins, 2016).

Consideration of the well-being of CMD patients is also important as a determining factor in practical decisions about the provision of treatment. If life with CMD is not worth living, this provides a strong reason against devoting significant healthcare resources to keeping these patients alive. CMD patients require 24-hour care, in addition to the costs of treating illnesses and infections often arising as a result of continued life-sustaining treatment. Expending these resources would be unjust if CMD patients are not gaining any benefit from continued life.

Contrary to the prevailing view in the bioethics literature, I think that the presumption that CMD patients do not have lives worth living is unjustified (Graham, 2017). Healthy people consistently underestimate the quality of life of those with severe disabilities. Moreover, the unfamiliarity of a condition like CMD makes it even more difficult to imagine how these patients are likely faring. Because intuitions about the well-being of those with severe disabilities are so often unreliable, I argue that the burden of proof is on those who claim that CMD patients do not have lives worth living, and that this burden of proof has not yet been satisfied. Given the lack of conclusive evidence that the lives of CMD patients are clearly worse than other severely disabled people who claim their lives are worth living, and given the unreliable nature of our intuitions regarding well-being and severe disability in general, I argue that we should allow that CMD patients sometimes have lives worth living and should not presume that they do not.

Why is the burden of proof on those who claim that CMD patients do not have lives worth living, rather than on those who suggest they could? When a particular course of action appears to involve positive harms, this generally provides a clear direction of burden of proof. In most cases, bringing about someone’s death, either through act or omission, harms them—especially in the absence of an expressed wish to be killed. Thus, we should assume that allowing CMD patients to die is the worst possible outcome until some contrary evidence can be provided. If it turns out that these patients are suffering to such an extent that their lives are not worth living, this will be an exception to the general rule that being killed is a harm. To meet this burden of proof, sufficient evidence must be presented that these patients do not have lives worth living.

I begin by providing a brief background on UWS and CMD. I then argue that the dissonance between healthy people and people with disability about the well-being of the latter should make us question our intuitions about patients with CMD. To evaluate their potential for lives worth living—and meet the burden of proof—we need to think carefully about what their lives are like, for them. Drawing on the existing neuroscientific literature, I develop an account of the cognitive capacities of CMD patients, to generate as clear a picture as possible of what their lives might be like. I then appeal to established philosophical theories of well-being to evaluate this kind of life. I conclude that the burden of proof is not met, and that it is far from clear that CMD patients do not have lives worth living. Next I outline the practical consequences of this conclusion.

## II. BACKGROUND: UNRESPONSIVE WAKEFULNESS SYNDROME AND COGNITIVE MOTOR DISSOCIATION

UWS/VS is one of several disorders of consciousness caused by severe traumatic or anoxic brain injury. Patients with UWS/VS undergo periods of wakefulness and sleep (i.e., eyes opening and closing), but they demonstrate no evidence of awareness of themselves or their environment, and no evidence of sustained, reproducible, purposeful, or voluntary behavioral response to stimuli (Multi-Society Task Force on PVS, 1994; Laureys et al., 2010).

A diagnosis of UWS/VS is made through a behavioral examination at the bedside, using an assessment tool such as the Coma Recovery Scale-Revised (Giacino, Kalmar, and Whyte, 2004). UWS/VS may be a relatively brief transitory state, with patients moving from coma, to UWS/VS, to the minimally conscious state (MCS), and in some cases making a good recovery. The longer a patient is UWS/VS, however, the less likely they are to recover consciousness. The Multi-Society Task Force on PVS regards they are “permanent” 1 year after traumatic brain injury, or 3 months after anoxic brain injury (Multi-Society Task Force on PVS, 1994).

Research over the last decade has shown that a minority of patients who consistently show no voluntary behavioral response to stimuli at the bedside are nevertheless aware. Using functional neuroimaging to monitor patient brain activity, researchers have been able to detect the volitional (i.e., non-reflexive) activation of specific brain areas in roughly 15% of patients diagnosed as UWS/VS (Monti et al., 2010; Cruse et al., 2011; Bender et al., 2015; Kondziella et al., 2016). Once a patient demonstrates evidence of consciousness using functional neuroimaging, they are considered CMD.^2^ Patients with CMD not only demonstrate a behavioral profile similar to UWS or MCS, but also demonstrate electrophysiological evidence of command following. Thus, a diagnosis of CMD allows for a spectrum of variability in the patient’s cognitive capacity; they may have a severe level of cognitive impairment, or very little (Schiff, 2015). Indeed, some CMD patients have been able to use functional neuroimaging to communicate with researchers (by answering yes-or-no questions), while others are only capable of command-following (Fernandez-Espejo and Owen, 2013).

The fact that CMD patients are covertly aware is morally significant, because it means that they satisfy a general condition for having subjective interests. In order for something to be good or bad for me (i.e., for it to contribute to or detract from my well-being), I must have a subjective point of view. Awareness seems to be a general condition for having such a perspective. The mental imagery task provides good evidence that CMD patients are capable of having certain mental states, which in turn provides sufficient warrant for ascribing to them the capacity for at least some kinds of subjective experience. In addition to simply being aware, however, CMD patients also retain a repertoire of cognitive capacities that exceed what we would expect of a patient diagnosed as UWS/VS.^3^

The presence of covert awareness in these patients—and thus, the capacity to suffer—has led several bioethicists to suggest that it may be in the best interests of CMD patients to be allowed to die (Kahane and Savulescu, 2009; Kitzinger and Kitzinger, 2013; Hawkins, 2016). Echoing this intuition, research suggests that a majority of healthcare professionals and members of the general public would not want to be kept alive in a disorder of consciousness like CMD. Demertzi et al. (2011) found that 82% of healthcare professionals would prefer not to be kept alive if they were in a UWS/VS, and 62% if they were “minimally conscious,” while Gipson et al. (2014) found that 64% of the general public would not want to be kept alive in a UWS/VS and 41.4% of respondents would prefer to be allowed to die if they were minimally conscious (although 36.4% of respondents said they were uncertain).

How can we explain these responses among health professionals and members of the public? On the one hand, perhaps they believe that patients with disorders of consciousness experience pain, or are suffering in other ways to such an extent that continuing to live in this state would be unbearable as a result. Alternatively, they might believe that continuing to live in a profoundly diminished state, where one can no longer pursue the things that had previously given one’s life purpose and in which one is completely dependent on others, is undignified and not worth living.

There are a few reasons we might question these intuitions. Healthy people tend to be poor at evaluating the quality of life of people with severe disabilities or illnesses. In a classic study, Albrecht and Devlieger (1999) found that many people with serious and persistent disabilities report a good or excellent quality of life, despite the fact that most external observers would rate these lives as undesirable, a phenomenon they describe as the “disability paradox.” This disparity between the reported quality of life of patients and the estimated quality of life of healthy people about similar circumstances has been observed across a range of health conditions, including cancer, stroke, arthritis, traumatic brain injury, cerebral palsy, and muscular dystrophy.

One possible explanation for the disability paradox is that people with disabilities simply misreport or exaggerate their well-being. Subjective scales of quality of life have little inherent meaning, because different people might mean different things when they say that their overall quality of life is “very good,” or that their overall health is “7 out of 10.” For example, research has shown that survey respondents spontaneously rate their overall health relative to a reference group of people roughly their own age, and also respond differently when they know they are being surveyed as a patient, rather than a member of the general public (Ubel et al., 2005). However, while some kinds of self-reports are susceptible to this sort of scale recalibration, research suggests that this cannot be the primary explanation for the discrepancy that exists between patients’ self-reports of quality of life and the imaginings of healthy people (Ubel et al., 2005).

Another possible explanation is that healthy people have difficulty predicting what it is like to have a disability and how it will affect their life. When imagining unfamiliar circumstances, such as living with a disability, people focus narrowly on those aspects of their lives that would be different; the so-called “focusing illusion.” As a result, they tend to overestimate the emotional impact of the disability (Schkade and Kahneman, 1998). Further, healthy people tend to underestimate the extent to which people with disabilities adapt to their condition. People with disabilities can adapt physically by adopting new strategies to accomplish tasks. They can also adapt psychologically by shifting their goals and priorities in life, finding meaning or purpose in new aspects of their lives, and even redefining for themselves what it means to be happy. However, research suggests that healthy people often underestimate their own ability to adapt to negative circumstances (Ubel et al., 2005).

This evidence suggests that the intuitions of healthy people about the quality of life of patients with severe health conditions may not be an accurate indication of how these patients are faring, even when the nature of their condition can be relatively well-described. Adding a further complication to the case of CMD patients is the fact that this condition is only just beginning to be described; we still know very little about what it is like to be in a state of disordered consciousness like CMD. Accordingly, it is possible that quality of life estimates of healthy people about CMD might be even more susceptible to confounds like a focusing illusion. While the physical limitations of these patients are clear, the ways in which these patients might adapt, or the ways in which their lives retain subjective value, will be less apparent. Even if healthy people were able to judge accurately the subjective quality of life of severely disabled people, the limited possible insight into the subjective experiences of CMD patients makes these judgments even more difficult and potentially less reliable.

Of course, the fact that many patients with severe disabilities report a good or excellent quality of life does not entail that patients with CMD would report the same if they were able to communicate. However, it does suggest that across a variety of severe disabilities and illnesses, patients are able to adapt to changing circumstances, including re-evaluating for themselves what is required for a good quality of life. Taken in conjunction with the relative unreliability of healthy people’s assessments of quality of life in disability, I argue that we cannot simply presume that CMD patients have a very low quality of life, and certainly not that their quality of life is so low that they would be better off dead. Indeed, even if it turns out that some people with disabilities overestimate their own quality of life, it is highly implausible that a person who claims to have a good quality of life would in reality be better off dead. We need to be open to the possibility that our intuitions about the quality of life of these patients can be mistaken.

The extent to which the well-being of CMD patients is satisfied or frustrated by their condition will depend on the kinds of prudential goods that remain open to them, which will in part depend on the cognitive capacities they retain. In the next section, I examine the current neuroscientific evidence for the cognitive capacities of CMD patients.

## III. THE COGNITIVE CAPACITIES OF CMD PATIENTS

### Command-Following to Detect Covert Awareness: The Mental Imagery Task

The standard paradigm for detecting awareness in behaviorally non-responsive patients, pioneered by Owen and colleagues (2006), used functional magnetic resonance imaging (fMRI) and involved a single patient who had been diagnosed as UWS/VS. This patient repeatedly demonstrated statistically significant levels of activity in the appropriate brain areas when instructed to imagine playing tennis or walking through her house for 30-second intervals. In both tasks, the patient’s brain activity was indistinguishable from that seen in healthy controls. Following these 30 seconds of activity, the patient was instructed to “relax,” whereupon brain activity ceased. After several repetitions of this task, researchers concluded that this patient was capable of understanding commands and voluntarily producing brain activity in response to those commands, and was thus aware.

A subsequent study by Monti et al. (2010) demonstrated that of 23 patients diagnosed as vegetative that were scanned, 4 (17%) were able to produce neural activation in response to command. Alternative command-following paradigms have used various imaging modalities, including electroencephalogram (Cruse et al., 2011; Gibson et al., 2016), positron emission tomography (Stender et al., 2014) and functional near-infrared spectroscopy (Kempney et al., 2016; Rupawala et al., 2018), to detect covert awareness in patients diagnosed as UWS/VS.

Moreover, some patients who are capable of performing mental imagery have also used this paradigm to communicate with researchers, by imagining certain tasks to answer “yes” or “no” to various questions. One patient, who had been presumed to be UWS/VS for 12 years, was able to correctly answer twelve different questions, across several imaging sessions, including their name, their location, the name of their personal care worker, and whether they were in pain (Fernandez-Espejo and Owen, 2013).

What conclusions can we draw about the cognitive capacities of CMD patients, based on the successful completion of the mental imagery task? At the very least, CMD patients are capable of sustained attention (required to maintain focus through each task), language comprehension (required to understand the instructions of the researchers), response selection (required to switch between alternative task requirements), decision-making and execution skills (required to decide whether to comply with the task instructions and carry out the mental task), and working memory (required to remember task instructions, and which task to perform). As Fernandez-Espejo and Owen (2013) argue, “these are all aspects of “top-down” cognitive control that are typically associated with normal levels of conscious awareness”.

The patient who was able to communicate with researchers demonstrates evidence of a number of further cognitive capacities. The ability to identify correctly one’s own name suggests self-identity, while the ability to identify correctly the year and his location suggests orientation in space and time. Further, the ability to identify correctly the name of his personal support worker, whom he had only met after his accident, suggests the capacity to form new memories. Finally, the ability to answer that he enjoyed watching hockey on TV, and that he was not in pain, demonstrates a capacity for personal preferences.

### Command Following to Detect Covert Awareness: Selective Attention Tasks

In a selective attention paradigm, participants are instructed to either attend to the audio presentation of a specific target word (e.g., “yes” or “no”) by counting the number of occurrences of that word, while ignoring distractors (e.g., numbers 1–9), or simply relax and pay no attention. Selective auditory attention tasks have been successfully used by patients with disorders of consciousness to demonstrate their covert awareness (Naci and Owen, 2013; Gibson et al., 2016). Additionally, two of these patients (one MCS and one UWS) were able to use selective attention to correctly communicate answers to biographical questions.

The selective attention task requires continuous monitoring and processing of auditory information and the filtering out of potential distractors. Selective attention is a foundational cognitive process that underlies more complex faculties like reasoning and information processing, and thus, the capacity for selective attention allows for the possibility that CMD patients retain the capacity for these more complex cognitive processes.

### Naturalistic Paradigms

Naci et al. (2014) measured the brain response of healthy participants as they viewed a short movie in the fMRI scanner. Healthy participants displayed highly synchronized brain activity in sensory-driven auditory and visual areas as well as in frontal and parietal regions known to support executive function. This result suggests that executive function in response to the movie drove brain activity in frontal and parietal regions, and, further, that the synchronization of this activity across individuals underpinned their similar subjective experience.

This same approach was then applied in two behaviorally non-responsive patients with unknown levels of consciousness. One patient, who had remained behaviorally non-responsive for a 16-year period prior to the fMRI scanning, demonstrated a highly similar brain response to the healthy participants, suggesting a similar experience of suspense during the movie. (This patient was subsequently determined to be CMD via an independent research scan.)

The capacity of CMD patients to understand complex, real-world narratives over time has recently been called “covert narrative capacity” and is supported by a sophisticated cognitive repertoire (Graham et al., 2016). First, covert narrative capacity implies the conscious processing of visual and auditory stimuli, including recognition of familiar objects, faces, and voices. Second, it demonstrates that patients retain executive function. Continuous engagement with a movie’s plot, including relating events in the film to one’s experience of the real world, which allows for making predictions about what will happen next, requires integrating auditory and visual information with one’s prior knowledge and experiences into a meaningful whole.

Third, demonstration of covert narrative capacity suggests a preserved “theory of mind,” the ability to infer and understand the mental states of others, as well as one’s own (Baron-Cohen, Leslie, and Frith, 1985; Henry et al., 2006). An important part of engaging with a complex narrative is making inferences about the mental states of the characters, in a variety of dynamically unfolding social contexts. Understanding and predicting the behavior of others requires accounting for their unique perspective of the world, as well as the state of the world from our own perspective, and differentiating between them.

Fourth, CMD patients with covert narrative capacity may have a preserved capacity to value. Participants demonstrated greater neural activation when the film’s protagonist pointed a gun at other people (e.g., a shopkeeper, a mailman), as compared to morally neutral objects (e.g., a mirror), and greater activation when the child pointed the gun at his mother, as compared to non-related characters.

Finally, covert narrative capacity suggests a preserved capacity for affective states, as well as the capacity for reflection. Suspense can elicit feelings of excitement, tension, anxiety, and anticipation. Research also suggests that the experience of suspense recruits brain regions involved in making strategic inferences, and involves future-directed cognitive processes (Chow et al., 2008; Lehne and Koelsch, 2015). Individuals experiencing suspense must have certain beliefs about the past as well as certain beliefs or expectations about the future and be capable of adjusting these beliefs when new information is presented. A CMD patient capable of engaging with a suspenseful stimulus is capable of making inferences about possible future states of affairs, and could also be capable of organizing her own experiences according to a temporally coherent structure.

### Research Using Facial Electromyography

A 2016 study by Fiaconni and Owen suggests that some CMD patients may be capable of experiencing humor. In their study, Fiaconni and Owen presented various joke and non-joke sentences to two patients diagnosed as UWS/VS, and measured their facial muscle response. The authors used facial electromyography (EMG)—which can detect subtle responses not possible through visual observation—to monitor activity in two facial muscle groups implicated in smiling and frowning: the *zygomaticus major* (smiling), and the *corrugator supercilii* (frowning) EMG. They argued that an observed increase in *zygomaticus* activity in response to jokes (i.e., a smiling response) would imply the preservation of the emotional response to humor.

In one patient, Fiaconni and Owen (2016) detected much higher EMG activity from the *zygomaticus* in jokes than non-jokes. This patient, like the healthy control group, also produced less EMG activity in the *corrugator* muscle group for jokes compared to non-jokes, confirming that the observed muscle activity did reflect a smiling response and not merely an increase in overall muscle activity. (This patient was also able to perform mental imagery in an independent experiment, and was thus CMD.)

This result suggests that a CMD patient can experience the emotional response to humor, in much the same way as healthy controls. Additionally, the cognitive requirements for joke understanding suggest several additional residual cognitive capacities in CMD patients understanding jokes.

Humor is based on the perception of incongruity. In the case of verbal or written jokes, it is the incongruity between what we expect based on the initial set-up of the joke, and the information conveyed in the punch-line. The set-up activates a certain “schema”—a dynamic mental representation—that allows us to make sense of the incoming information. Then, the punch-line introduces information inconsistent with the original schema, forcing us to reinterpret this information and search for a different schema that can make sense of it. This simultaneous activation of two incompatible schemas generates the incongruity we find enjoyable or amusing, and is the essence of humor.

Thus, joke understanding requires highly sophisticated language comprehension, working-memory, long-term memory, and executive function. First, listeners must integrate the information conveyed in the set-up of the joke and activate an appropriate schema in working memory. They must then retrieve background information from long-term memory to reinterpret the information conveyed in the set-up and combine this with the information already being held in working memory, to generate a new schema (Moran et al., 2003). In verbal jokes based on semantic ambiguity (e.g., puns), listeners must maintain both schemas in working memory and exercise differential phonological processing over and above what is required in processing non-humorous language (Goel and Dolan, 2001). Similarly, the comprehension of verbal jokes in which the ambiguity can be resolved by the punch line requires executive function to integrate new information with prior knowledge (Samson et al., 2009).

### Research Investigating the Experience of Physical Pleasure and Pain

Several studies have attempted to investigate the possibility that patients with UWS/VS can experience pain (Laureys et al., 2002; Boly et al., 2005; Kassubek et al., 2003). The classic view of our basic pain system comprises two largely segregated subsystems, referred to as the “pain matrix”: the lateral neuronal network and the medial neuronal network. These networks correspond to the sensory-discriminative and affective-motivational dimensions of pain. Studies have demonstrated that UWS/VS patients may display brain activation in areas associated with the experience of pain, but these areas of activation are disconnected from each other (Laureys et al., 2002; Boly et al., 2005; Kassubek et al., 2003). This makes it unlikely that patients are consciously aware of the painful stimuli, and thus, unlikely that they experience the negative affect—suffering— which is typically associated with pain experience. In contrast, patients in the MCS are believed to be capable of consciously experiencing pain (Boly et al., 2008; Schnakers et al., 2010; Chatelle et al., 2014), and thus, of experiencing suffering from physical pain.

Given this evidence, it is likely that CMD patients can experience pain (Graham et al., 2018). Intact functional connectivity between primary and associative cortices appears to be a critical component of conscious awareness, and the conscious experience of pain. Thus, the fact that CMD patients retain sufficient functional connectivity between primary and associative cortices to support awareness suggests that they may also retain the capacity for pain experience. This relationship between the presence of awareness and the ability to consciously experience pain is supported by the capacity for pain in MCS patients. Research has also demonstrated that many patients diagnosed as UWS/VS respond to the pain cries of others (Yu et al., 2013).

A similar argument can be made for the capacity for pleasure in CMD patients. The experience of pleasure in the human brain relies on the activation of “hedonic hotspots” in the basal forebrain. If these brain areas remain functionally intact in CMD, this would provide evidence that these patients can experience pleasure. When patients recover from coma to UWS/VS or MCS, they recover function in the basal forebrain, which explains their recovery of sustained spontaneous eye-opening. Accordingly, if damage to the basal forebrain is not sufficient to abolish wakefulness in CMD patients, it may also be the case that the brain mechanisms underlying pleasure causation in the basal forebrain are preserved as well. Moreover, research has shown that connectivity within the default mode network is closely correlated to the level of consciousness of brain-amaged patients (Vanhaudenhuyse et al., 2010). This network of brain regions has also been speculated to be involved in the subjective experience of pleasure (Kringelbach and Berridge, 2009). Given this relationship between connectivity of the default mode network and level of consciousness, we would expect that CMD patients would retain a degree of connectivity of the default mode network. If this is the case, it lends support to the idea that these patients retain the capacity for the subjective experience of pleasure.

### The Residual Cognitive Capacities of CMD Patients and the Capacity for Well-Being

Empirical research strongly suggests that the residual cognitive capacities of CMD patients go beyond merely retaining awareness (table 1). While we cannot say for certain how these patients experience the world, it seems plausible that they have some sense of their previous lives, can experience some degree of emotion, and can think about and evaluate at least some aspects of their current and future condition. This is likely to lead many to conclude that these patients are suffering a great deal. What do I mean by suffering in this context? Roughly, I take suffering to be the intensely felt, negative, affective experience of loss (or absence) of that which is constitutive of one’s well-being. Suffering is mental, emotional, and spiritual, and the cause and intensity of our suffering depends on the things that we value. Prolonged and intense pain can lead to suffering, insofar as it restricts or deprives us of that which we find important and valuable in life. However, just as pain need not lead to suffering, suffering can exist in the absence of pain, such as in the grief of a parent who has lost a child.

**Table 1.** The residual cognitive capacities of CMD patients

Research type	Capacity implicated (+conscious awareness)	Example studies
Mental imagery task	Language comprehension, working memory, attention, response selection, decision-making	Owen et al. (2006); Monti et al. (2010); Cruse et al. (2011); Bardin et al. (2011); Chennu et al. (2013); Stender et al. (2014); Edlow et al. (2017)
Selective attention task	Monitoring of information, filtering out distractors, selective attention	Naci and Owen (2013); Gibson et al. (2016)
Communication (via mental imagery/selective attention)	Self-identity, orientation in space and time (self-consciousness), formation of new memories, personal preferences	Monti et al. (2010); Fernandez, Espejo, and Owen (2013); Naci and Owen (2013)
Naturalistic paradigms	Language comprehension, recognition of faces, executive function, theory of mind (possibly capacity for emotions/affective states, capacity to value, psychological connectedness to future states of self)	Naci et al. (2014, 2018); Naci, Sinai, and Owen (2017); Haugg et al. (2018)
Facial electromyography	High-level language comprehension, short-term memory, long-term memory, executive function, affective states	Fiaconni and Owen (2016)
Neurophysiology of pain/pleasure experience, emotion-related cognition	Negative affective response to pain (i.e., suffering), positive affective response to physical pleasure (i.e., liking), affective response to the pain of others.	Laureys (2002); Boly (2005); Boly (2008); Schnakers (2010); Kassubek (2003); Yu et al. (2013); Pan et al. (2018)

CMD patients can no longer access much of what contributes to well-being, and even after their awareness has been discovered, the inability to communicate renders them largely isolated from those around them. As a result, we might think that these patients suffer *even worse* than patients with UWS/VS, because of the sophisticated cognitive capacities they retain. Rather than existing in a state of unconsciousness—insulating patients from the experience of suffering—CMD patients have the capacity to suffer and may in fact suffer in a variety of ways. Conversely, the sophisticated cognitive capacities these patients retain might also allow them to experience various kinds of pleasure and enjoyment, or other prudential goods. In the next section, I consider three prominent theories of prudential value and examine to what extent an appeal to these theories can meet the burden of proof that CMD patients do not have lives worth living.

### Hedonism

Consider first a hedonist account of well-being. Hedonism understands prudential value as consisting of pleasant experiences and the absence of painful experiences. Something contributes to my well-being insofar as it causes me to experience pleasure, and detracts from my well-being insofar as it causes me to feel pain. CMD patients may experience physical pain. The care and symptom management required by CMD patients is extensive and may include skin injuries and pressure ulcers, articular deformations, muscle spasticity, nutrition, hydration, and deglutition problems, respiratory and cardiovascular issues, intracranial pressure, as well as the rigors of daily living (e.g., hygiene, bathing, feeding, or dressing). Any of these issues might lead to significant discomfort or pain. Indeed, the fact that these patients may retain a sense of themselves in space and time, as well as short-term and long-term memory, makes the management of physical pain even more important. Numerous studies have shown that anticipating pain, or being anxious about pain, can exacerbate the pain experienced (Edwards et al., 2006; Tracey and Mantyh, 2007).

CMD patients might also be capable of pain beyond the merely sensory. Emotional or mental states may be experienced as unpleasant or painful—as causing suffering—and would thereby detract from a patient’s well-being. Given their capacity for various affective states, combined with the emotional or behavioral disturbances that often accompany significant brain injury (e.g., delusions, severe mood disturbance, agitation, confusion), it is possible that CMD patients may experience significant emotional distress. Indeed, evidence from other patient populations with similar rapid-onset loss of motor function and capacity for communication (e.g., Guillain-Barré syndrome) suggests that extended periods in an intensive care environment may result in psychotic symptoms, including hallucinations, as well as anxiety or depression (Weiss et al., 2002).

Further, the capacity for language comprehension, combined with the inability to functionally communicate without neuroimaging, may cause CMD patients to feel isolated. Their ability to focus their attention and comprehend language suggests that they may be capable of following conversation happening around them. The feeling that one is being ignored or marginalized, or that one is “invisible,” may be a source of suffering. Similarly, the capacity of CMD patients to ascribe thoughts to others (i.e., to exercise a “theory of mind”), as well as to experience their distress, may allow CMD patients to comprehend the emotional suffering of others, including their family members. This could lead to patients experiencing feelings of sadness or guilt.

However, many potential sources of physical pain are contingent on the degree of care that CMD patients receive. Although the kinds of interventions they require place them at increased risk of developing illness, which may cause suffering, the use of analgesics to manage acute or chronic pain and attentive care on the part of caregivers can minimize their physical pain. Similarly, the knowledge of caregivers that these patients are aware may in itself help to reduce some of the potential emotional or mental pain they experience, by allowing caregivers to change their behaviors (Graham, 2015). Knowing that a CMD patient is aware can make it easier for caregivers to interact with patients, and may help to reduce a patient’s unpleasant feelings of isolation or loneliness.

CMD patients are also likely to be capable of experiencing physical pleasure. Many of the same sensory pleasures which are available to healthy individuals (e.g., listening to a favorite piece of music or watching an exciting sports game, the feeling of a gentle massage or a warm breeze on a sunny afternoon) are available to CMD patients, while other physical comforts and adequate stimulation can help to provide a positive balance of pleasant experiences. On a hedonist account of well-being, provided their pleasant experiences outweigh their unpleasant experiences to a sufficient degree, CMD patients may be capable of faring reasonably well. Indeed, while the sources of pleasure open to these patients may be narrower than that of a healthy individual, it may also be the case that many of the sources of suffering which might frustrate the well-being of healthy people would not affect these patients.

A critic of hedonist views of well-being might object that while the experience of pleasure and avoidance of pain are certainly important to overall well-being, they are not entirely constitutive of well-being. Simply having one’s basic needs met—in a way that maximizes pleasure and minimizes pain—may be insufficient to make life worth living for an individual with cognitive capacities at the level of CMD patients, because the range of pleasures available may be too narrow or because more than just pleasure (and the absence of pain) has prudential value. One CMD patient, with whom neuroimaging-based communication was possible, repeatedly stated that he was not in pain and that he still enjoyed watching hockey on television. While encouraging, these responses provide only a small window into the hedonic experiences of CMD patients. Because the subjective experience of CMD patients remains unclear, consideration of their experiential interests presents an uncertain account of their well-being, but not enough to justify the claim that their lives are not worth living.

### Desire-Satisfaction

According to desire-satisfaction theories of well-being, my well-being consists in getting what I desire; my life goes better for me to the extent that my desires are satisfied and worse to the extent that my desires are frustrated. The subjective nature of desire-satisfaction theories is viewed by many as appealing, because it supports the intuition that what is good for persons should be in some way attractive to them. However, its subjective nature makes it particularly difficult to apply to non-communicative patients, like those with CMD. Evidence from functional neuroimaging suggests that CMD patients retain awareness of themselves in space and time, that they are capable of forming new memories, and that they are capable of integrating new information from their environment with past knowledge retrieved from long-term memory. It is possible that CMD patients may have desires and preferences about future states of affairs, including desires about themselves. Without a robust means of communicating with patients, however, it is difficult to know what these desires are. And, while some CMD patients have communicated with researchers by answering yes-or-no questions through mental imagery, it would be impractical to use fMRI to interrogate all the desires a CMD patient might have, given the limited availability of fMRI and the coarseness of response which patients can give.

One possible alternative is to appeal to the desires CMD patients had prior to their injury, to determine their current well-being. If patients’ previously held desires are frustrated by their injury, it follows that these patients are less well-off than if those desires had been satisfied. Given the ways in which the lives of CMD patients are limited by their physical and cognitive deficits, it seems plausible that many (if not most) of their prior desires will be frustrated by their condition. For example, the inability to satisfy long-term goals like success in one’s career, travelling to a new country, or learning a musical instrument, detract from a person’s well-being. It also seems likely that the inability to continue satisfying the day-to-day desires healthy people take for granted would negatively impact CMD patients. The inability to move by themselves and the resulting disruption to autonomy is likely to undermine a wide range of immediate desires like the desire to feed, wash, and dress oneself, scratch an itch, go for a walk, read a book, chat with one’s partner, or work in the garden. The accumulation of these more immediate desires being continually frustrated could severely detract from well-being.

However, it is not obvious why failing to satisfy a past desire necessarily detracts from one’s well-being, unless one continues to hold the desire. Suppose as a child I wanted to eat ice cream every day once I became an adult. But satisfying this desire as an adult will not enhance my well-being, if I no longer desire to eat ice cream every day, even though doing so would satisfy a desire I held in the past. Unless we have reason to think that patients with CMD continue to hold the desires they held prior to their injury, or more specifically, continue to assign the same prudential weight to their satisfaction, the fact that these past desires go unsatisfied does not seem to negatively impact their well-being.

Is there any evidence to suggest that CMD patients might no longer hold the desires they did prior to their injury? First, it may be the case that because of their injuries, CMD patients simply are not capable of having the same kind of complex desires as healthy people. Deficits in the cognitive functions required to represent and reason about certain states of affairs may restrict CMD patients to more rudimentary desires (e.g., avoiding pain, and experiencing pleasure). However, the evidence discussed above suggests that CMD patients may be capable of more complex desires (e.g., the desire to achieve a meaningful goal).

There are other reasons for thinking that the desires of CMD patients change post-injury. When confronted with a severe injury or disease, patients can accommodate their condition by undergoing a process known as “response shift” (Sprangers and Schwartz, 1999; Rapkin and Schwartz, 2004; McClimans et al., 2013; Blome and Augustin, 2015). This process involves changing one’s internal standards, values, and conception of quality of life. Response shift may function as a coping mechanism, helping to buffer individuals from the social and emotional consequences of their condition. They may adopt values and goals that are more attainable or change their standards for evaluating their own well-being to accommodate their illness. For example, those diagnosed with terminal cancer might undergo a shift in their values away from career achievement, towards meaningful relationships with family. Because they no longer desire career success, the fact that these past desires go unsatisfied would not impact their well-being.

The potential for significant adaptation to a debilitating condition is exemplified by patients with locked-in syndrome. In the classic syndrome, patients are incapable of movement or verbal communication, apart from vertical eye movement, but remain fully conscious. We might expect that locked-in patients would be more likely to be suffering than even CMD patients. These individuals are unable to pursue most of the desires and personal projects of their pre-injury life and appear cut off from most of the goods which are generally thought to make a human life worth living (e.g., developing deep personal relationships, knowledge, achievement, and developing one’s talents). Moreover, they are fully aware of the ways that their well-being is frustrated, whereas the cognitive limitations of CMD patients may temper the complexity of their suffering. It seems plausible that if any individual would find continued existence distressing in a way that would cause significant suffering, it would be locked-in patients.

Nevertheless, several studies have shown that the self-reported quality of life of locked-in patients is within the same general range as that of healthy individuals (Laureys et al., 2005; Lule et al., 2009; Bruno et al., 2011). Bruno et al. (2011) surveyed a group of 65 patients with locked-in syndrome, on various aspects of their quality of life. Seventy-two percent of respondents indicated that they were happy, while 82% of respondents were satisfied with their personal relationships with others. However, researchers also reported that only 21% of respondents were engaged most of the day in activities they considered “important,” while 12% of respondents were dissatisfied with their participation in recreational activities, and 40% dissatisfied with their social participation. Moreover, 58% said they would not want to be resuscitated in the event of cardiac arrest, while 7% expressed a wish for euthanasia (Bruno et al., 2011).

A related study by Rousseau et al. (2015) measured changes in self-reported quality of life of a sample of 39 patients with locked-in syndrome, between an initial survey in 2007, and a second survey in 2013. They found that nearly 75% of patients reported a stable or improved quality of life over the 6 years and that degree of physical handicap in 2007 was not correlated to quality of life in 2013. Moreover, they found that patients who exhibited an objective decline in their health condition (e.g., new needs for gastrostomy, tracheostomy, urinary probe, or new reports of chronic pain) did not exhibit a significant difference in their quality of life compared to those patients whose physical condition remained unchanged. Interestingly, they found that of the 23 patients who wished for resuscitation if needed in 2007, 15 maintained this preference in 2013, while the one patient who wished for euthanasia in 2007 no longer did so in 2013.

One way of interpreting these findings is that these individuals have undergone a change in their values, goals, and desires, to accommodate the challenges imposed by their condition. While it is safe to assume that these individuals previously had desires that were inconsistent with being locked-in, the fact that they report a reasonably high quality of life in the present suggests that the frustration of these past desires has not had an overwhelming impact on their current well-being, because these past desires are no longer held or no longer viewed with the same importance. For example, I might rate my satisfaction with my social relationships as a 1 out of 5, 6 months after my injury, but rate it as a 3 out of 5 after 12 months, simply because I have changed my values and expectations, and not because any aspect of my social relationships has changed. A shift in values and expectations is likely to lead to a change in desires as well, given that what I desire is largely determined by what I value. While the experience of locked-in patients may not be analogous to the experience of CMD patients, reports of good quality of life in locked-in patients illustrate how people may undergo a significant shift in what matters to their well-being, and demonstrates how healthy people might misjudge whether such a life is worth living.

One might object that the occurrence of response shift is essentially a case of an “adaptive preference,” a preference formed in response to a deprived set of options which does not reflect an individual’s “true” interests (Elster, 1983; Nussbaum, 1992). For example, people who claim to prefer to remain in abusive relationships, or who claim to be satisfied in oppressive societies are typically cited as paradigm examples of holding an adaptive preference. We would not want to say that these people are faring well, even though they claim that their desires are satisfied. On many accounts, adaptive preferences are problematic insofar as they are irrational, resulting from causal processes that are non-autonomous (Elster, 1983), or are justified by factors that do not actually support the adaptive preference (Bovens, 1992). Thus, we should not take adaptive preferences as indicative of an individual’s well-being.

However, there seem to be many preferences we form in response to a restricted set of options, but which we would not think are irrational, and whose satisfaction would contribute to our well-being. Bruckner (2009) argues that there should be a presumption in favor of adaptive preferences, but this presumption can be defeated when an agent fails to reflectively endorse this preference. Suppose I have a life-long desire to play professional golf and practice constantly for many years. While I eventually become very good, I will never be good enough to play professionally. I then adjust my desire, and determine that it would really be best for me to become a golf instructor, and compete in amateur tournaments. After reflecting on this change, I am glad I have adopted this new desire, rather than continuing to be frustrated by my inability to achieve my previous desire. What is important is that were I to reflect on my preference, I would endorse it as something I genuinely desire; I am not just “fooling myself.” On a desire-satisfaction account, I am faring better because I can now satisfy my desire. And this seems consistent with the intuition that I really am better off, having changed my desire in response to my circumstances, rather than continuing to be frustrated in the pursuit of something I cannot achieve.

Many cases of response shift could be understood as this sort of adaptive preference change, where a patient reflectively endorses a new preference in response to a restricted set of options. The occurrence of response shift in a wide range of severe health conditions —cancer, stroke, organ transplant, AIDS, multiple sclerosis, locked-in syndrome—suggests that it may be possible in CMD patients as well (Schwartz et al., 2006). If CMD patients can change their values and desires in response to their circumstances, they could avoid the desire frustration that would compromise their well-being.

Unfortunately, there is very little direct evidence for whether CMD patients undergo response shift, and thus, whether they might fare reasonably well on a desire-satisfaction account of well-being. In any case, the degree to which CMD patients are able to adjust to their condition is likely to depend on a range of factors, including the level of care they receive, family support, and their own personality. Some patients are more resilient than others and are better equipped to adjust to a catastrophic injury or illness, and there is no reason to expect CMD patients to be any different. Nevertheless, a desire satisfaction account does not clearly support the presumption that CMD patients do not have lives worth living.

### Objective Goods

Hedonist and desire-satisfaction accounts of well-being provide an incomplete picture of the well-being of CMD patients. Because we have limited insight into the experiences, desires, and values that constitute well-being on these kinds of accounts, it is difficult to specify to what extent they are faring well. For objective accounts of well-being, however, this is less of a problem. Objective-list theories pick out certain goods, like knowledge, achievement, happiness, and friendship, which contribute to our well-being, but need not appeal to an individual’s attitudes or values to explain the prudential value of these goods. On this sort of view, knowledge makes me better off, whether I value knowledge or not.^4^ Similarly, perfectionist views of well-being hold that what makes something constitutive of well-being is that it helps to perfect human nature or develop distinctly human capacities.

At first glance, CMD patients appear to be paradigm cases of impoverished lives according to objective theories of well-being. Their physical and cognitive limitations render them incapable of achieving personally meaningful goals or projects, developing their talents, or having deep personal relationships. While they may be capable of experiencing sensory pleasure, more meaningful or “higher” pleasures are largely unavailable to them. Their complete dependence on others and the resulting impact on their social roles and sense of self may deeply compromise their self-respect. Similarly, Richard Kraut argues that what is good for human beings is to flourish as human beings, by developing and exercising their physical, cognitive, sensory, social, and affective powers. The capacities and powers of CMD patients are highly circumscribed by their condition, which implies that these individuals are faring very poorly; their lives diverge too severely from “the shape that a human life should have” (Kraut, 2009, 170).

I think this assessment is too quick. It is perfectly consistent to argue that CMD patients are made worse off by their injury because they are prevented from possessing certain objective goods, but that their lives are still worth living overall. Evaluating whether the life of a CMD patient is worth living must weigh the objective goods that they lack against those goods they retain, the objective ills that they possess, and the objective ills they lack.

Consider again patients with locked-in syndrome. Even if we think that these patients might overestimate the quality of their lives, those who report being happy surely have lives worth living. Are CMD patients so much worse off than locked-in patients that locked-in patients have lives worth living, while CMD patients do not? I do not think this category distinction is justified.

First, the objective goods available to locked-in patients are likely to be similarly circumscribed as patients with CMD. In both cases, a lack of motor function severely constrains individual agency, the ability to pursue one’s goals and projects, and the extent to which one can influence the external world. The inability to perform even the simple tasks of daily life without complete dependence on others could potentially be a source of deep frustration. A dramatic shift in one’s social role, as well as one’s physical capacities, could also be highly disruptive to a patient’s sense of self, in both CMD and locked-in syndrome. Similarly, complications from the need for medical interventions like tracheotomy or gastronomy are likely to cause physical discomfort in both cases. And yet, many locked-in patients report a good quality of life.

Second, CMD patients may still be capable of some objective goods. I argued previously that CMD patients can experience pleasure and pain, which means that a favorable balance of sensory pleasure (and absence of pain) may contribute to their well-being on an objective account. Their ability to understand language, develop new memories, and exercise executive functioning suggests that they may be capable of gaining knowledge, another commonly cited objective good. Derek Parfit has suggested that “awareness of beauty” may be an objective good, and the perceptual and cognitive capacities of CMD patients gives no reason to think that they could not possess this objective good as well (Parfit, 1984).

What about objective goods like achievement? An influential account has been given by Gwen Bradford, who argues that achievement is composed of a process and a product, where the process is difficult, and competently causes the product (Bradford, 2015). Given their circumstances, most tasks for patients with CMD are difficult. Bradford gives an example of a one-armed person being able to tie shoelaces as an achievement, given its difficulty. Analogously, performing mental imagery and using it to communicate could be considered an achievement for CMD patients. Still, it is not clear that this kind of achievement in and of itself would contribute much to their well-being. When we think of achievements in terms of their contribution to our well-being, we tend to think of tasks requiring sustained effort over a significant period of time, and which are somehow meaningful or valuable (e.g., training for and running a marathon, writing a screenplay, raising a child). The physical limitations of CMD patients would seem to make it virtually impossible for them to competently cause a product like this, and thus, to achieve something that would significantly contribute to their well-being.

However, we might understand the product of achievement in a less restrictive way. Specifically, if the product of the achievement is a state of themselves, perhaps CMD patients could still achieve it, despite their physical limitations. For example, we might think that the extent of their injuries would make it very easy for CMD patients to become angry, bitter, or depressed people. Thus, it might be very difficult at times to remain positive and maintain a desire to continue living in this kind of state. Yet, patients who successfully complete the mental imagery task presumably have the desire to let those around them know that they are aware (or else they could simply ignore the task instructions). The ability to remain positive in the face of tremendous adversity (including the will to engage with the outside world) could be understood as a kind of achievement, one which CMD patients must continually accomplish.

The above examples suggest that CMD patients may be capable of possessing some objective goods after all, and thus, that their lives may not be so impoverished as to not be worth living. Similarly, they may be capable of a degree of flourishing, insofar as they are able to exercise their cognitive, sensory, social, and affective powers (in the ways I have described throughout), albeit to a limited degree. While this may not be an ideal human life on an account like Kraut’s, it may still be one worth living.

One obvious difference between CMD and locked-in patients, however, is the ability to communicate. “Low-tech” communication devices, such as alphabet boards, allow locked-in patients to communicate through blinks or eye movements, with the help of a second person. More advanced communication devices, such as infrared eye movement sensors or cameras which track eye movement, can allow locked-in patients to spell out words on a computer, and communicate via the internet (Lule et al., 2009). Conversely, not all CMD patients have the capacity to communicate using functional neuroimaging, and of those that do, opportunities for communication in this way are infrequent and currently limited to yes-or-no responses.

It has been suggested that this difference in communicative capacity can account for why locked-in patients have lives worth living and CMD patients do not (Kahane and Savulescu, 2009; Hawkins, 2016). In locked-in patients, the capacity to communicate is critical to well-being insofar as it allows patients to participate in social and family life, and maintain relationships with the people around them. Even if communication is arduous, it protects patients form the kind of isolation that we might think incompatible with a life worth living.

Nevertheless, I argue that even in the absence of an ability to communicate, CMD patients can continue to have meaningful relationships with others and through their presence be involved, at least to an extent, in family and community life. Specifically, CMD patients can maintain the kind of relationships that exist between those who care, and those who are cared for.

Eva Feder Kittay argues that human dignity stems from our capacity to care for and be cared for by others, that “one gives care because of its intrinsic worth—and the only thing worthy of such efforts is another who in and of her/himself has intrinsic value” (Kittay, 2005). Even in relationships in which one party is completely dependent on another, the provision of care indicates an affective bond, and investment in another’s well-being: “when we acknowledge how dependence on another saves us from isolation and provides the connections to another that makes life worthwhile, we can start the process of embracing needed dependencies” (Kittay, 2011). Dependence is understood not in opposition to independence, but rather in opposition to isolation; dependence can create a positive connectedness with others. This connectedness is not reliant on the capacity to reciprocate care, or even to communicate with one’s caregivers. While CMD patients may be unable to communicate their desires or preferences, and those who care for them cannot be sure exactly what they understand or experience, the attitude of care and respect on the part of the caregiver, and the receipt and experience of care and respect by the care-receiver, constitutes a meaningful relationship for both parties.

I suggest that the capacity to experience being valued and cared for can ground meaningful relationships for CMD patients, relationships with prudential value. The residual cognitive capacities demonstrated by CMD patients—focusing attention, understanding language, experiencing affective states, and demonstrating a theory of mind—suggest a capacity to appreciate the care of others and ascribe meaning to their actions. These sorts of caring relationships have many of the sorts of features that we would ascribe to objectively good relationships: trust, respect, mutual valuing, and affection. If CMD patients retain the capacity for emotions and theory of mind, they might continue to benefit from being a part of the lives of those around them, even if their role in these relationships has changed. Even in the absence of communication, these patients can still be present in the lives of others and share experiences with them, and their presence can provide prudential value both for the patient, and for those around them. In fact, research suggests that the most powerful predictor for good quality of life in locked-in patients is perceived social support (Lule et al., 2009; Rousseau et al., 2013). While these relationships may be less rich and complex than the kinds of relationships they might have had pre-injury, they can still have prudential value.

## IV. CONCLUSION

CMD patients may retain significantly greater capacities than their lack of behavioral responsivity suggests. This means that an accounting of their well-being is much more complex than previously thought, and we may need to rethink our assumptions that the well-being of these patients is necessarily poor, or that their lives are not worth living. I have argued that on a hedonist, desire-satisfaction, or objective account of well-being, it remains possible for these patients to possess a passable level of well-being. The presumption that these patients do not have lives worth living is not justified by the available evidence.

Of course, it does not follow from my argument that CMD patients *necessarily* have lives worth living. Whether these patients do in fact realize their potential for well-being is a separate question, and may be largely dependent on the quality of the care they receive. If a CMD patient is able to continue to participate in family life, is comfortable and free of pain, and receives adequate stimulation commensurate with their cognitive capacities, I think this can be a life worth living. Patients like Scott Routley and Jeff Tremblay, who were able to demonstrate covert awareness using functional neuroimaging, exemplify this kind of life (Lunau, 2014). Unfortunately, for many patients diagnosed as UWS/VS, this is not what their lives are like. These patients languish in long-term care facilities, receiving little stimulation and potentially in pain from lack of movement. The few prudential goods these patients possess may not be enough to outweigh the prudential ills which accompany a complete loss of autonomy and capacity to communicate. The fact that patients are aware is not sufficient to make their lives worth living. Indeed, even if patients are able to communicate using functional neuroimaging, it does not follow that their lives are worth living, if these patients do not possess sufficient prudential goods. With the right care, CMD patients can possess these goods, and have lives worth living.

I have argued that the claim that the lives of CMD patients are prudentially neutral (because they do not experience enough to have well-being) is false, and the claim that their lives are prudentially bad is not sufficiently justified. However, it is a further question whether there is an obligation to expend significant health care resources to keep these patients alive. Even if we accept that these patients may benefit to some degree from continued life, we might think that they do not benefit enough to justify the substantial cost of their long-term care (approximately £90,000 per year, or $115,000 USD) (Formby, Cookson, and Halliday, 2015). In this case, the requirements of beneficence are in tension with the requirements of justice to fairly allocate scarce health resources. I will not attempt to defend this resource expenditure here. What my argument demonstrates is that one cannot simply argue that spending these resources is unjust on the grounds that CMD patients do not receive *any* benefit. There is a real tension between beneficence and justice, which will require careful consideration and informed debate. Understanding what it might be like to be a patient with CMD and how this may differ from our intuitions is critical to this discussion, and this is what I have set out in this paper.

Finally, it is critical that physicians and surrogate decision-makers consider the potential for well-being and suffering in CMD patients when making decisions about pursuing life-sustaining treatment in the early stages after brain injury. Prognosis after severe brain injury is highly uncertain, and in many cases decisions are made to allow patients to die well before a reliable prognosis can be determined. These decisions are often motivated by a worry that patients will become “trapped” in a prolonged disorder of consciousness, with no way of ending their lives once they reach a point of being physiologically stable. In order to avoid this outcome, patients are allowed to die before being given a real chance to recover. I do not think it is necessary or appropriate to provide life-sustaining treatment to all severely brain injured patients, when it is sufficiently clear that they will not recover to an acceptable level. However, in order to make an informed decision about what is in the best interests of patients with disorders of consciousness, surrogate decision-makers and physicians need to consider what life might actually be like for them, and how they will respond to this kind of life. In the case of CMD patients, this may still be a life worth living, and a life worth preserving.

## Footnotes

^1.^
Throughout this paper, I use the terms “well-being,” “quality of life,” and “life worth living.” “Quality of life” is typically used in the social sciences and health care, and reflects an empirical measure of how a person’s life is going for them, either at a certain time, or globally, across a range of domains. “Well-being” is more common in the philosophical literature, to refer to what is non-instrumentally good for a person, whereas a “life worth living” is a rough threshold of well-being below which persons are faring so poorly that continued life is no longer in their interests. While “well-being” and “quality of life” are both conceptualized and assessed in domain-specific ways, and thus are not strictly speaking equivalent, I understand them as ultimately being concerned with the same thing, namely, how well a person’s life is going for that person.

^2.^
Because functional neuroimaging has not yet been incorporated into routine diagnosis of disorders of consciousness, patients are not officially diagnosed as CMD even after demonstrating covert awareness. For the purposes of this discussion, CMD patients are those patients who demonstrate evidence of awareness (e.g., command following) through functional neuroimaging, but remain behaviorally non-responsive at the bedside.

^3.^
There is debate in the bioethics literature concerning whether or not UWS/VS patients, and even MCS patients, have moral status (i.e., for their interests to matter morally) (Brody, 1992; Levy and Savulescu, 2009; McMahan, 2009; Braddock, 2017). These arguments generally turn on whether UWS/VS patients meet a threshold for moral status, such as the possession of certain cognitive capacities (e.g., sentience, self-consciousness). Without committing to a particular account of moral status here, I submit that on any plausible account, CMD patients meet the threshold for moral status, given their residual cognitive capacities.

^4.^
For this reason, it has been argued that hedonism is really just a very short objective list theory, with pleasure being the only objective good (Fletcher, 2013).
